# Psychosocial and Socioeconomic Patterns of Post-Rehabilitation Reintegration Among Former Substance Users in China: An Exploratory Multiple Correspondence Analysis of Administrative Case Records

**DOI:** 10.3390/bs16081307

**Published:** 2026-08-01

**Authors:** Yunyi Xiao, Hongyi Lin, Miguel Ribeiro Ramos, Paul Montgomery-Marks

**Affiliations:** 1 School of Politics and Law, Hanshan Normal University, Chaozhou 521041, China; 2 School of Social Policy, University of Birmingham, Birmingham B15 2TT, UK

**Keywords:** China, family relationships, multiple correspondence analysis, post-rehabilitation reintegration, psychosocial disadvantage, relapse vulnerability, social support, substance use

## Abstract

Background: Relapse risks and social reintegration after substance rehabilitation are not only influenced by the individual’s social context, but also by social, family, and socioeconomic circumstances. However, there is limited quantitative research that has studied the co-occurrence of these factors among persons who have been rehabilitated from substance dependence in the Chinese context. Methods: Administrative case narratives from the Legal Services Casebook Database of the Ministry of Justice of China from 2017 to 2021 were analysed. Of the 227 drug rehabilitation-related cases initially identified, 92 contained available and codable information for all variables required for Multiple Correspondence Analysis. Narrative data were coded and then re-categorised into indicators of age, gender, educational level, family relationships, social relationships, financial status, employment status, and recorded relapse history. MCA was not used to estimate the causal effects or prevalence of these categories across the nation, but rather as a method for exploring patterns of association. Results: For the retained complete-case subsample, characteristics of lower educational attainment, unstable employment, financial difficulty, poor family relationships, and poor social relationships tended to be positioned close together in the MCA space. This suggests a pattern of co-occurring psychosocial and socioeconomic disadvantage within the analysed records. Relapse history made a weak contribution to the main MCA dimensions, indicating that recorded relapse alone did not organise the principal structure of association in the data. Conclusions: The findings offer exploratory evidence that reintegration-related disadvantage in these records was configured across relational and socioeconomic domains. Caution should be used when interpreting the results, as they are from a selected, small, complete-case subsample and from variables recoded from administrative narratives. Future research should utilise larger-scale, longitudinal, and mixed-methods datasets to conduct validation studies, to investigate whether these findings are consistent with the remaining data, and to examine their association with long-term reintegration outcomes.

## 1. Introduction

Social reintegration following substance rehabilitation is not only a social issue, but also a behavioural and psychosocial process. Individual abstinence is not the only key to sustained recovery; family support, employment opportunities, community-based supports, and social welfare resources play a role in sustained recovery.

In China, post-rehabilitation reintegration has gained more significance in the overall background of socioeconomic change. While there has been significant progress in living conditions and access to education and healthcare, as well as social security, in recent decades (Liu et al., 2023), rehabilitated former substance users could still be faced with barriers once they leave formal rehabilitation or supervision settings. Such barriers include poor employment stability, low education levels, poor family dynamics, low social support, and ongoing stigma. These conditions could limit people’s ability to create viable lives even in a society that is undergoing material progress.

Previous research on substance use has shown that recovery is closely linked to employment and socioeconomic stability, whilst vulnerability, family circumstances, and the wider socio-ecological context are associated with relapse (Laudet, 2012; Yang et al., 2015). Unemployment, the lack of family support, and poor social relationships, in particular, may be factors that leave individuals vulnerable during the reintegration process. Rapid social change and population movement have led to new patterns of social relations in the Chinese context but could also have the effect of eroding social support and community bonds (Xu et al., 2022; Yang et al., 2015). These changes necessitate consideration of the interaction between social, familial, educational, and economic factors in the lives of rehabilitated former substance users.

This study thus focuses on how different forms of disadvantage appear together in case records of rehabilitated former substance users in China. This study does not view substance use or relapse as an isolated event, but instead explores the association between multiple categorical variables, including education, employment, financial status, family relationships, social relationships, and relapse history. In this way, this study provides a descriptive account of how selected relational and socioeconomic disadvantages co-occur in administrative case records, and what these patterns may suggest about the context of post-rehabilitation support.

In this study, social reintegration was not assessed directly as an outcome or a longitudinal process. Instead, it was approached indirectly through categorical indicators recorded in administrative case records, including demographic, relational, socioeconomic, and relapse history information.

### 1.1. Psychosocial and Socioeconomic Factors in Substance Use Recovery

The literature shows that individual characteristics and the social environment in which they live can affect post-rehabilitation outcomes. Age, gender, and educational status have been identified as important correlates of substance use and recovery. Young adulthood (18–45 years) is a time of transition and vulnerability, as people negotiate transition situations around work, family, and identity development (Satre et al., 2012). Young people may also face more challenges following rehabilitation, with some studies showing that younger drug users are at a higher risk of relapsing (Andersson et al., 2019). The gendered patterns are also noticeable. Males may be more likely to be exposed to high-risk social contexts, while females may have unique recovery issues stemming from unemployment, having children, and unstable relationships (Jiang et al., 2015; Zhou et al., 2017). Educational disadvantage also heightens vulnerability because it negatively affects a person’s ability to gain stable employment and their risk of social marginalisation (Kendler et al., 2018; Horwood et al., 2010).

These individual characteristics are intricately linked with relational and structural conditions. Poor social support and interpersonal networks may result in limited access to emotional, informational and practical support, which may make individuals more vulnerable during recovery (Xu et al., 2022; Yang et al., 2015). Relationships with family members are similarly important. Stable and supportive family environments can support recovery, while family conflict, emotional neglect, and family disruption can exacerbate psychological distress and hinder reintegration (Broekhof et al., 2023; Gerra et al., 2020; Hardaway & Cornelius, 2014; Azmi et al., 2018; Suwanchatchai et al., 2024). These challenges can be exacerbated by economic hardship, financial insecurity and low employment, which can hinder people’s ability to establish stable everyday routines and reintegrate into society (Yang et al., 2015; Baptiste-Roberts & Hossain, 2018). Employment is therefore not only essential as a source of income, but also a significant pathway to restoring social integration, the normal routines of daily life and personal stability. Employment is linked to better recovery outcomes and reduced risk of relapse as demonstrated in previous research (Huang et al., 2021; Laudet, 2012).

There is also a close connection between structural and relational conditions and psychological and behavioural processes in reintegration. There are several common underlying issues that many substance users encounter, such as trauma, low self-esteem, poor resilience, anxiety, loneliness, and depression, which can all be problematic during recovery (Wang et al., 2023). After rehabilitation, individuals might also experience embarrassment, loneliness, and worries about the future. Furthermore, the continued presence of environments or social networks with which they were associated with substance-using behaviours may maintain substance use behaviours, especially if community-based supervision and support are not adequate (Shen et al., 2018). However, community-based supervision systems in China face limitations due to coordination and resources, which may diminish their ability to offer ongoing post-rehabilitation support (Gu & Zhang, 2018).

Overall, the literature indicates that reintegration needs to be read not solely as an issue of risk factors. Rather, social, family, school, economic, and psychological factors can interact and reinforce each other. This requires consideration of the extent to which specific factors relate to recovery and the extent to which they co-occur as sets of factors and patterns of advantage and disadvantage.

### 1.2. Research Gap and Present Study

While prior research has focused on demographic, social, and economic characteristics linked to substance use and relapse, several gaps remain. First, although a considerable amount of research exists on various risk factors or outcomes, less research has been performed on how the various dimensions of disadvantage are co-occurring in the lives of rehabilitated former substance users. Low educational attainment, unemployment, financial insecurity, weak social relationships, and limited family support do not necessarily function independently in real-life reintegration processes. Instead, they may form interconnected patterns, influencing the chances for recovery and social engagement of individuals.

Second, quantitative evidence is still sparse, particularly within the Chinese context, based on administrative or case data reporting these configurational patterns. Administrative case records created by the official record system can give systematically recorded data on the demographics, family situation, social relations, employment, financial situation, and relapse history across case narratives. Although not collected for the purposes of empirical social research, data such as these can provide structured and comparable information across cases that can be useful for social research (Evans et al., 2010).

However, such variables are not always well adapted to the traditional regression type of analysis, particularly when attempting to identify patterns rather than estimate causality. This is especially true for administrative data with a case-based structure that contains a high proportion of categorical variables and for which the associations between variables can be multidimensional, rather than linear.

Based on these gaps, this study has used Multiple Correspondence Analysis (MCA) to analyse administrative case records from the Legal Services Casebook Database in China. MCA is an exploratory method that is applicable to categorical data and is helpful in finding patterns of association between several variables. In contrast to the independent influence of one variable on another, MCA allows the researcher to understand how categories are placed in relation to each other as well as whether certain types of disadvantage tend to cluster together.

This study examines the associations among age, gender, level of education, family relationships, social relationships, financial status, employment status and history of relapse among rehabilitated former substance users. It is not intended to make causal inferences or to generate nationally generalisable estimates, but instead to give an explorative description of the social and socioeconomic contexts that are found in administrative case records. Through this approach, the study extends the literature on the issues of substance use recovery and social reintegration by moving beyond the focus on individual risk factors to a wider perspective of multiple disadvantages in the Chinese context.

## 2. Methods

### 2.1. Data Source

The data for this study were drawn from administrative case records from the Legal Services Casebook Database of the Ministry of Justice of China (China Ministry of Justice, 2017–2021, 2024). The database holds case-based records that are gathered through the judicial administration system and include documented information on individuals undergoing substance rehabilitation or post-rehabilitation reintegration.

The records contain socio-demographic data (age, gender, education level, etc.) and data relating to rehabilitation and reintegration (type of intervention, duration of treatment, adherence description, employment, family, social relationships, finances, and relapse). Therefore, these data are an empirical resource to explore the recording of various social, relational, and socioeconomic conditions in relation to the reintegration of rehabilitated former substance users.

The database is an administrative data source that gives relatively standardised and systematically recorded case information. Administrative data have been identified as useful resources for studies on substance use and rehabilitation, because they enable researchers to explore trends in reported cases, while also requiring attention to data completeness and reporting consistency (Evans et al., 2010). More recent debates about addiction-related data have also highlighted the need for structured, accessible and reusable data for enhanced research transparency and comparability (Sixto-Costoya et al., 2025). Case records can also contain information about the processes of rehabilitation and recovery over time, which can be useful for identifying patterns of recovery, but were not used in the present study to make causal claims about the processes of recovery over time (Dennis et al., 2003).

The Legal Services Casebook Database was not used in this study to make prevalence estimates or to draw causal inferences, but to examine associations between casebook categories in administrative case narratives. This is in line with the exploratory nature of the study, which aims to see the way in which the various demographic, social, relational and socioeconomic characteristics are distributed in the case records at hand.

### 2.2. Sampling and Inclusion Criteria

A primary search of the ‘Legal Services Casebook Database’ revealed that there were 227 cases relating to drug rehabilitation between 2017 and 2021. This timeframe was chosen because it includes relatively recent administrative documents on the practice and support for rehabilitation in China. This study utilised Chinese terminology related to drug rehabilitation to conduct a keyword search in the ‘Legal Services Case Database’ in order to identify relevant cases. The retrieved records were subsequently evaluated, with particular attention paid to whether their content related to rehabilitation or reintegration into society following rehabilitation, and whether they contained codable information on the variables required for MCA. It should be noted that this database comprises a collection of administrative case records and does not constitute a population-level database covering all rehabilitated former substance users in China.

Cases were screened using the inclusion criteria. Each case eligible for inclusion in the analysis had to contain sufficient codable information on the key variables for Multiple Correspondence Analysis (MCA) analyses: age, gender, educational level, family relationships, social relationships, financial status, employment status and relapse history. Records were excluded if they lacked data or where data for one or more of these variables were ambiguous or could not be categorised consistently.

Of those that were screened, 92 were kept for analysis. While the sample of identified cases was larger than the final sample for analysis, the objective of the study was to investigate associations between categorical social, demographic, relationship, and economic factors. For this reason, only those cases that had a high level of completeness and comparability were kept to ensure that there was interpretive consistency within the MCA framework.

A limiting factor is the change in the number of identified cases from 227 to 92 analysed cases. Cases which were excluded due to lack of information, ambiguous or unclassifiable information could differ in systematic ways from the cases included, thus introducing the potential for selection bias. The retained sample should, therefore, be viewed as a complete-case analytical subsample as opposed to a sample of all rehabilitated former substance users in China. Therefore, these results should be regarded as exploratory, pattern-based findings rather than statistically representative. The case screening process is summarised in Table 1.

Exclusion reasons were not treated as mutually exclusive, because a single record could lack codable information on more than one MCA variable. Therefore, excluded records were grouped as cases with missing, ambiguous, or uncodable information on one or more core variables. This comprehensive case screening may have introduced selection bias, as the cases included may differ from those excluded in terms of the level of detail in case records, case complexity, institutional follow-up, or disease severity.

### 2.3. Coding Procedure, Case Screening, and Data Preparation

Ethical permission for this study was obtained from the University of Birmingham ethics committee before the data were collected and analysed. The study used secondary administrative data from the Legal Services Casebook Database of the Ministry of Justice of China. As the data were based on case records generated through the judicial administrative system, the original material consisted primarily of descriptive case narratives rather than pre-structured quantitative variables.

The coding was carried out in accordance with a pre-defined coding scheme, which was established before the MCA. The coding was based directly on the original Chinese administrative narrative data, and no translation was undertaken before coding. The initial coding was carried out by the first author, who read through each case narrative to extract information regarding age, gender, education, employment, financial status, family relationships, social relationships and relapse history. Interpretive variables (family relationship, social relationship and financial status) were coded, with rules drawn up to differentiate between favourable and unfavourable categories. A co-author coded a subsample of these cases to maintain consistency, focusing especially on these variables of interpretation. Ambiguous or borderline cases were discussed between authors, and repeated readings of the original administrative narratives resolved cases. Cases that could not be coded consistently were not forced into categories and were excluded from the complete-case MCA sample. As the coding review was used as a consistency-checking procedure rather than as a formal reliability study, no inter-coder reliability statistic was calculated. Coding was given priority to information which pertained to the rehabilitation or post-rehabilitation period, where possible. However, because the administrative narratives did not always provide standardised timing for each variable, the coded variables should be understood as case attributes rather than temporally ordered measures.

Then, the data of retained cases were recoded to structured categorical variables for quantitative analysis of the relevant information. These variables included demographic variables, relational factors, socioeconomic factors, as well as information on relapse history. Detailed operational definitions are given in Section 2.4 below.

The administrative case data referred to in this study have been categorised to suit the present statistical analysis. As these data were not originally collected for research purposes, coding decisions were made cautiously and were undertaken only where sufficient information was provided in the case descriptions. The final analytical sample did not include cases where information was missing, ambiguous, or unable to be classified on any one of the core variables.

All variables were recoded as categorical variables prior to MCA. These comprised demographic factors (age group, gender, education level), relational factors (family relationship, social relationship), socioeconomic factors (financial status, employment status), and reintegration-related outcomes (relapse history). These variables were chosen due to their applicability to post-rehabilitation reintegration and for the ability to analyse them as categorical indicators.

### 2.4. Measures

#### 2.4.1. Age

Age information was obtained from the case narratives and recoded into two categories: 18–45 years old (0) and 46–69 years old (1). This categorisation reflects life-stage differences in substance use patterns and rehabilitation needs, as previous studies have suggested that age is associated with different risks, social responsibilities, and recovery trajectories (Yu & Zhang, 2023; Substance Abuse and Mental Health Services Administration [SAMHSA], 2020). Cases with missing or unclear age information were excluded.

#### 2.4.2. Gender

Gender was coded solely if it was explicitly or clearly mentioned in the case narrative, for example, if there was a direct reference to sex or gender. This was recoded as male (0) or female (1); cases where there was missing information on gender were not included. Past research has shown gender differences in patterns of substance use, treatment experiences, and recovery outcomes; thus, gender is a demographic variable of interest (He et al., 2013; Grella et al., 2003).

#### 2.4.3. Educational Level

Educational level was extracted from the case descriptions and grouped into two categories: college-level education or above (0) and below college-level education (1). This distinction aims to reflect the phenomenon of educational stratification, which has been shown to be associated with vulnerability to substance use, employment opportunities, and social reintegration outcomes (Pan, 2021; Kendler et al., 2018).

#### 2.4.4. Social Relationships

Social relationships were coded as good (1) or poor (0) based on descriptions of social assistance, interpersonal contact, and broader social interaction. This binary classification captures the presence or absence of meaningful social connections, which previous research has linked to health, social support, and reintegration outcomes (Umberson & Karas Montez, 2010; Xu et al., 2022).

#### 2.4.5. Family Relationships

Family relationships were extracted from narrative case information and coded as good (1) or poor (0). Following García-Montes et al. (2009), good family relationships were understood as involving stable family structure, emotional support, or financial support. Poor family relationships referred to family conflict, family disintegration, emotional neglect, or lack of support. Cases lacking adequate family relationship information were excluded.

#### 2.4.6. Relapse

To assess the presence of relapse, a binary variable was created: at least one recorded relapse (1) or no recorded relapse (0), based on information in the case records. No recorded relapse should not be interpreted as evidence of no actual relapse; it means that in the available administrative narrative, there was no evidence of recorded relapse. Because previous substance use research considers relapse to be an important post-treatment or post-rehabilitation concern, it was included as one post-rehabilitation-related indicator (Nuwara & Hayajneh, 2022).

#### 2.4.7. Financial Status

Financial status was categorised as stable (1) or difficult (0), based on whether the case narrative suggested that the individual was able to meet basic living needs without severe financial pressure. This measure reflects a broader understanding of economic stability that is not limited to income alone but also includes perceived or documented economic hardship in everyday life (Wahler & Otis, 2014).

#### 2.4.8. Employment Status

Employment status was recoded as employed or self-employed (1) and unemployed or with unstable employment (0), based on the description in the case narrative. This should be considered a general measure of labour-market attachment, not a standardised measure of employment stability. Employment was added as previous literature has demonstrated that work and participation in the labour market can be beneficial for recovery as it helps to normalise their daily habits, reestablish their position in society and reduce the risk of relapse (Laudet, 2012; Augustyn et al., 2020).

### 2.5. Data Storage and Confidentiality

All data used in this study were securely stored in an approved institutional data management system in accordance with relevant data protection requirements. Access to the dataset was restricted to authorised members of the research team.

As this study relied on secondary administrative data, all information was processed in a de-identified form to protect confidentiality. Data storage and management procedures complied with relevant ethical guidelines and data protection regulations, including the General Data Protection Regulation (GDPR).

### 2.6. Data Analysis

Data analysis was conducted using IBM SPSS Statistics, version 30.0, with Multiple Correspondence Analysis (MCA) as the main method of analysis. MCA is especially appropriate for the analysis of relationships between categorical variables and for visualising patterns of association in multidimensional data.

To explore the relationship between demographic, relational, socioeconomic and reintegration-related variables (age, gender, educational level, family relations, social relations, financial status, employment status, and relapse history), MCA was used. The aim was to ascertain if certain categories were clustered together in the multidimensional space and if there were some general patterns of reintegration-related advantage or disadvantage.

MCA was chosen because all the central variables were categorical variables, and the intention of the study was to discover configurations of association and not to predict one dependent variable. MCA can be used to explore which categorical variables are co-occurring and to investigate profiles when compared to regression-based methods, which calculate the independent effects of predictors on an outcome. Thus, it is suitable for analysing the presence of combinations of family support, education, employment, financial status and social relationships across coded administrative cases.

Therefore, it is not possible to interpret the analysis as causal. The goal was not to evaluate hypotheses or make inferences at the population level, but to discover relational patterns in the administrative case records that are retained. The retained sample is suitable for analysis as it contains complete and comparable categorical data across all cases, but it should not be considered statistically representative of all rehabilitated former substance users in China.

The eight variables were used as active variables in the MCA. The first two dimensions were kept since they had the highest eigenvalues and the most easily interpretable structure. The variables were discriminated by determining which variables contributed most strongly to each dimension, and the proximity of the categories in the joint plot was interpreted as co-occurrence rather than as formal clusters of cases or individuals.

## 3. Results

### 3.1. Descriptive Characteristics of the Sample

Firstly, descriptive statistics were used to explore the distribution of the key variables in the analytical sample used (see Table 2). Most cases were coded as male (72.8%), while 27.2% were coded as female. Most cases were coded as involving individuals aged 18–45 (88.0%), with a minority in the 46–69 age group.

There was a high proportion of educational disadvantage in the sample. A large majority of cases were coded as involving below college-level education (80.4%), whereas 19.6% were coded as involving college-level education or above. Family relationships were relatively evenly distributed, with 53.3% being coded as good family relationships and 46.7% being coded as poor family relationships.

Social relationships showed a more disadvantaged pattern. In total, 64.1% of cases were coded as having poor social relationships, while 35.9% were coded as having good social relationships. As regards the history of relapse, 30.4% of cases had at least one documented relapse, whilst 69.6% had no record of relapse.

A high socioeconomic vulnerability was also present in the sample. A total of 54.3% of cases were categorised as financially difficult, and 45.7% were categorised as financially stable. Regarding employment, 34.8% were employed or self-employed, while 65.2% were unemployed or in unstable employment.

Overall, the descriptive data suggest that the retained sample was low educated, had poor social relations, financial hardship, and poor employment stability. Many cases in the retained sample recorded psychosocial and socioeconomic challenges relevant to post-rehabilitation reintegration.

### 3.2. Multiple Correspondence Analysis

Multiple Correspondence Analysis was conducted to examine patterns of association among the categorical variables. The joint plot generated in SPSS provides a visual representation of the relationships among variable categories in a two-dimensional space. On the plot, each point represents a category, and the relative distance between categories indicates their pattern of association. Categories positioned close to one another are more likely to co-occur in the sample, while categories positioned further apart are less likely to be associated (Greenacre, 2017).

The first two dimensions were retained for interpretation because they represent the major structure of association in the data. These are not to be interpreted as separate variables. Instead, they are considered to be larger underlying structures that are generated by the simultaneous distribution of several categorical variables. The meaning of each dimension thus depends on a process of identification of the variables and categories that strongly drive each axis (Husson & Josse, 2014).

The contribution of each of the variables to the two dimensions was determined by discrimination measures (see Table 3). In Dimension 1, the strongest contributions were made by social relationships (0.612), family relationships (0.559), education (0.447), and financial status (0.540). There was also a moderate contribution from employment status (0.345) and a weak contribution from relapse history for this dimension (0.005). These results indicate that Dimension 1 is more indicative of a general pattern of psychosocial and socioeconomic disadvantage.

The highest contributions in Dimension 2 were for gender (0.469) and age (0.420). Employment status also made a moderate contribution to Dimension 1 (0.345). This indicates that Dimension 2 is largely related to demographic differentiation, and not necessarily to the general psychosocial and socioeconomic structure that is reflected in Dimension 1.

The first two dimensions explained 45.82% of the reported variance. They were retained for the main visual interpretation because they had the highest eigenvalues and provided the clearest interpretable two-dimensional structure, although they did not fully summarise the dataset. Consequently, these dimensions were used for interpretation (see Table 4). In this study, the focus of the interpretation lies in the substantive meaning of these two dimensions, as the research interest lies not merely in the statistical simplification of the data but rather in the patterns of association. However, the spatial proximity of categories should not be interpreted as evidence of causal relationships.

In the retained complete-case subsample, the MCA results indicated that the social, family, educational, financial, and employment-related categories were the most influential, with scores contributing the most to the primary dimension of association. The overall MCA structure was not strongly organised by relapse history, by contrast.

### 3.3. Psychosocial and Socioeconomic Patterns of Reintegration

**Figure 1:**
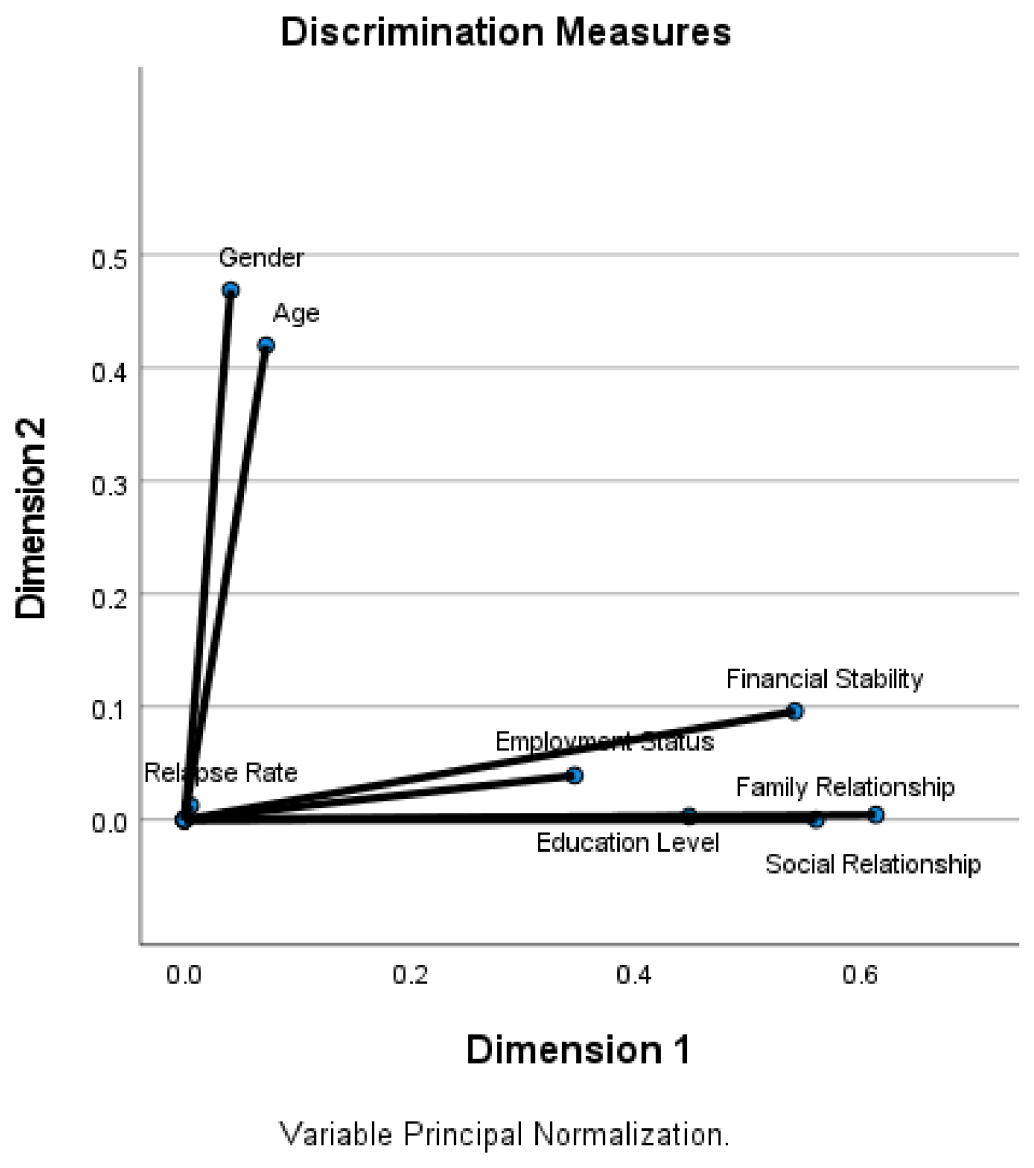
Joint plot of category points from the multiple correspondence analysis.

The joint plot shows the distribution of the categories on the two dimensions that were kept (see Figure 1). At the positive end of Dimension 1, categories indicating below college-level education, unemployed or unstable employment, financial difficulty, poor family relationships, and poor social relationships were positioned close together.

Note. The variable label “Relapse Rate” in the SPSS-generated plot refers to relapse history, coded as at least one recorded relapse or no recorded relapse. Other category labels correspond to the recoded variables described in Table 2 and Section 2.4.

This suggests an exploratory pattern of co-occurring disadvantage among some coded cases in the retained sample. In opposition, categories of college-level education or above, employed or self-employed status, financial stability, and good family and social relationships were placed on the other end of Dimension 1. This opposition indicates that Dimension 1 is related to differences between reintegration conditions that are relatively disadvantaged and reintegration conditions that are relatively stable after rehabilitation.

Family relationships and social relationships were located near each other, suggesting that these realms of relationships were correlated in the data structure. Similarly, financial status and employment status were represented in a related spatial niche, indicating that there was a socioeconomic pattern.

Demographic variables, including age and gender, were more highly related to Dimension 2. This means that the demographic differentiation was a secondary structure somewhat independent from the psychosocial and socioeconomic pattern represented by Dimension 1.

Based on the MCA results, the MCA space seemed to be imbalanced with regard to the disadvantage-related categories. Instead, poor family relationships and poor social relationships tended to cluster with lower educational attainment, unstable employment, and financial difficulty. This pattern is consistent with the assumption that reintegration-related conditions may have overlapping psychosocial and socioeconomic aspects but without causal interpretation due to the exploratory nature of the design.

## 4. Discussion

### 4.1. Main Findings

The study explored patterns of association among the selected variables of rehabilitated former substance users in China using Multiple Correspondence Analysis. The results indicate that psychosocial and socioeconomic factors, especially with respect to social relationships, family relationships, educational level, financial status, and employment status, were the strongest contributors to the central MCA dimension. On the other hand, age and gender were more strongly associated with the second dimension.

The results indicate that relational and socioeconomic conditions may be interrelated in the retained administrative cases, rather than operating as isolated characteristics. There was an association between poor social relationships, poor family support, low educational attainment, unstable employment and financial difficulties, suggesting a pattern of compounding disadvantage. However, relatively stable family, social, educational and economic circumstances seemed to create a contrasting pattern of relative reintegration stability.

These findings are important from the behavioural science point of view, as they indicate that recovery and reintegration cannot be solely explained by relapse behaviours or individual motivations. Instead, they are located within a complex socio-psychological context, such as family support and background, social relationships, employment stability, financial security, and educational background.

### 4.2. Psychosocial Disadvantage and Behavioural Reintegration

The findings are consistent with the notion that reintegration processes can have several psychosocial and behavioural aspects. Conditions in the family, social, and economic support may vary related to reintegration. This separation is crucial as reintegration barriers often do not exist in isolation. Rather, they might overlap and reinforce each other.

These results agree with the previous studies. Previous research has indicated that lower educational attainment is linked to a higher risk for substance use and to negative social outcomes (Kendler et al., 2018). Family relationships are also widely known to be a protective factor in the recovery process, with supportive families supporting the recovery process and reducing the likelihood of relapses, whereas family conflict or a lack of family support can negatively affect the recovery process (García-Montes et al., 2009; Zhu et al., 2018).

The role of social relationships in this study can also be understood through the concept of social capital (Putnam, 2000). Interpersonal networks may provide emotional support, practical assistance, access to resources, and a sense of belonging. Previous studies have similarly shown that stronger social relationships are associated with more favourable rehabilitation outcomes and a lower risk of relapse (Granfield & Cloud, 2001; Laudet et al., 2009).

Economic factors are also part of this more extensive pattern. Financial resources and employment are significant not only for the material stability they provide, but also for an individual’s ability to reestablish their daily routines, social roles, and participation in the normal life of the community. This aligns with research from around the world indicating that employment and economic security are significant factors in long-term recovery (Nagelhout et al., 2017; Compton et al., 2014).

Overall, the findings suggest that social, relational and socioeconomic conditions may be relevant to behavioural reintegration after drug rehabilitation. That implies that vulnerability to relapse and stability in recovery may need to be understood in relation to the broader social and relational contexts recorded in the administrative cases.

### 4.3. Family, Social Support, and Relapse Vulnerability

An interesting finding is that the history of relapse was only slightly loaded on the primary MCA dimensions. This should not be taken as a sign that relapse is not significant. Instead, it might be related to the fact that administrative relapse measures are not always sensitive enough to fully capture the broader nature of post-rehabilitation recovery and stability of behaviours. A combination of factors such as family support, stable employment, social network, psychological vulnerability and exposure to environments associated with previous substance use may be relevant to relapse vulnerability, although these relationships were not directly tested in the present MCA.

The administrative dataset used in this study does not include standardised psychometric measures. However, this does not imply that psychological processes do not play a significant role. Previous research suggests that there may be pathways linking psychological factors to adverse social and interpersonal circumstances. For instance, the tendency to relapse is influenced by family functioning, self-esteem, and resilience in individuals with substance use disorder (SUD) (Xia et al., 2022). Social support has also been associated with motivation for abstinence and access to self-control in the context of Chinese male substance users (Xu et al., 2022). Anxiety, loneliness, drug craving, and depression have been found to be closely interconnected during rehabilitation (Chen et al., 2021).

Thus, the negative factors emerging from the MCA, including poor family relationships, weak social relationships, unstable employment, and financial difficulty, may be seen as social negative factors, but also as factors which can further add to psychological vulnerability. These patterns indicate a possible interplay between individual behavioural processes and psychosocial environments in which recovery occurs, which may affect relapse vulnerability.

Future studies should include psychological and behavioural measures along with administrative, survey-, and interview-based measures. This type of research would support a better understanding of the interplay among family functioning, social support, self-esteem, resilience, self-control, emotional distress, and structural disadvantages that contribute to the risk of relapse and to successful reintegration over the long term.

### 4.4. Implications for Behavioural and Psychosocial Intervention

The results have implications for behavioural and psychosocial interventions after drug rehabilitation. Although this study did not examine specific intervention models, the findings support consideration of post-rehabilitation support that addresses family functioning, social isolation, employment instability, financial pressures, mental health, and community reintegration.

Service providers may need to offer more than one type of service to people affected by multiple disadvantages, such as vocational guidance, family relationship work, psychological support, social network rebuilding, and community reintegration services. If an individual’s difficulties are relatively limited, or if their family or financial circumstances are relatively stable, they may require only a lower level of support or services. However, if they are facing multiple difficulties, they may require more long-term or coordinated case management.

The results also indicate that rehabilitated former substance users cannot be assumed to be a homogeneous group. Rather, post-rehabilitation support may need to be differentiated according to each person’s configuration of disadvantage. Some people may need support for employment alone; others may need family mediation, social support, psychological counselling, or financial support. People facing multiple risks of disadvantage may need support in a number of areas.

These implications should be interpreted as preliminary and hypothesis-generating. The present study did not test the effectiveness of any intervention model. Rather, it suggests that future research and practice may benefit from considering relapse prevention alongside the relational and socioeconomic challenges recorded in the administrative cases.

### 4.5. Limitations and Future Research

Several limitations should be acknowledged. Firstly, of the 227 identified cases, only 92 were included in the final analysis, as the MCA requires complete and codable data for all core categorical variables. This reduction may have affected the representativeness of the sample analysed. This comprehensive case screening may have introduced selection bias, as the included cases may differ from those excluded in terms of the level of detail in case records, case complexity, institutional follow-up or disease severity. Consequently, the study findings should be regarded as exploratory in nature and should not be generalised as definitive estimates.

Second, this study used administrative case records that were not originally produced for academic research. As a result, the coding and interpretation of certain variables may have been influenced by variation in reporting quality, record completeness, and the level of detail provided in individual case narratives. Furthermore, to perform MCA, a number of complex variables were simplified into binary categories. This coding strategy improved comparability across cases but may have reduced the richness of the original administrative narratives and obscured certain intermediate situations, such as partial family support, unstable but ongoing employment, or mixed social relationships.

Although a co-author reviewed a subset of coded cases, the coding procedure was not designed as a formal independent double-coding reliability study. Future research should use independent double coding and report inter-rater agreement statistics.

Third, the study relied on cross-sectional administrative data and was thus unable to draw causal inferences or to study changes over time. Some case records may have contained data on the recovery process, but the present analysis was not planned as a longitudinal study.

Fourth, the data did not contain standardised measures for mental health, stigma, self-esteem, resilience, self-control, drug craving or perceived social support measures. This hindered the study’s capacity to explore psychological and behavioural processes potentially influencing reintegration because of structural and relational disadvantage.

Finally, the sampling period (2017–2021) coincided with the COVID-19 pandemic and the initial phase of the pandemic in China, characterised by more stringent restrictions. Disruptions due to the pandemic may have impacted employment opportunities, mobility, access to community-based supports, and the operations of local supervision and rehabilitation services. Thus, the particular social and institutional situation during this time period may have exacerbated reintegration problems, as noted in this study.

Future research should employ larger and more diverse samples, longitudinal study designs, and methods that draw on mixed data sources. Future studies that combine administrative and survey data with psychological assessments, behavioural indicators and qualitative interviews may help examine whether different forms of disadvantage overlap and how they relate to relapse risk, behavioural stability and long-term reintegration trajectories. Future studies could also report additional MCA interpretation statistics, such as category contributions, quality of representation and cos^2^ values, or compare MCA findings with other exploratory approaches such as latent class analysis or cluster analysis.

## 5. Conclusions

Multiple Correspondence Analysis was used to examine associations among demographic, relational, socioeconomic and reintegration-related variables in administrative case records of rehabilitated former substance users in China. The results indicated that in the retained complete-case subsample, the categories were not evenly distributed along the MCA space with regard to disadvantage. In particular, lower educational attainment, unstable employment, financial pressures, poor family relationships, and poor social relationships tended to be positioned close together, suggesting an exploratory pattern of co-occurring psychosocial and socioeconomic disadvantage.

This study provides exploratory evidence on psychosocial and socioeconomic patterns recorded in post-rehabilitation administrative case narratives within the Chinese context. The results suggest that future reintegration support may need to consider broader relational and socioeconomic factors alongside relapse prevention and individual behavioural support.

Although the findings are exploratory and based on a selected complete-case subsample of records, the results point to the potential value of differentiated and coordinated support for rehabilitated former substance users. Future studies should use larger, longitudinal, and mixed-methods designs to examine how psychological vulnerability, behavioural processes, service access, and long-term reintegration outcomes interact over time. These findings should not be interpreted as proof of causal relationships, longitudinal reintegration trajectories, or population-level patterns, as the analysis is based on a selected, complete-case subsample and on variables coded from the administrative narratives.

## Figures and Tables

**Table 1:** *Case screening process.*

Screening Stage	Number of Records
Initially identified drug rehabilitation-related records	227
Excluded due to missing, ambiguous, or uncodable information on one or more MCA variables	135
Final complete-case analytical sample	92

**Table 2:** *Descriptive characteristics of the retained sample.*

		Frequency	Percent
**Gender**	Male	67	72.8
	Female	25	27.2
**Age**	18–45	81	88.0
	46–69	11	12.0
**Education Level**	College-level education or above	18	19.6
	Below college-level education	74	80.4
**Family Relationship**	Good family relationships	49	53.3
	Poor family relationships	43	46.7
**Social Relationship**	Good social relationships	33	35.9
	Poor social relationships	59	64.1
**Relapse** **History**	At least one recorded relapse	28	30.4
	No recorded relapse	64	69.6
**Financial Stability**	Financially stable	42	45.7
	Financial difficulty	50	54.3
**Employment Status**	Employed or self-employed	32	34.8
	Unemployed or unstable employment	60	65.2
	**Total**	92	100.0

**Table 3:** *Discrimination measures from MCA.*

Discrimination Measures			
	Dimension		Mean
	1	2	
Gender	0.041	**0.469**	0.255
Age	0.072	**0.420**	0.246
Education Level	**0.447**	0.003	0.225
Family Relationship	**0.559**	0.000	0.280
Social Relationship	**0.612**	0.004	0.308
Relapse History	0.005	0.013	0.009
Financial Stability	**0.540**	0.096	0.318
Employment Status	**0.345**	0.039	0.192
Active Total	2.622	1.043	1.833
% of Variance	32.777	13.038	22.908

Note. Numbers in bold denote the key factors of each dimension; Discrimination values above 0.4 are considered moderate to high.

**Table 4:** *Eigenvalues from MCA.*

Dimension	Eigenvalue	% of Variance
1	2.622	32.78
2	1.043	13.04
3	1.018	12.73
4	0.941	11.76
5	0.820	10.25
6	0.650	8.13
7	0.520	6.50
8	0.385	4.81

## Data Availability

The data that support the findings of this study are available from the corresponding author upon reasonable request.
